# Homogeneous and Narrow Bandwidth of Spike Initiation in Rat L1 Cortical Interneurons

**DOI:** 10.3389/fncel.2020.00118

**Published:** 2020-06-17

**Authors:** Stefano Borda Bossana, Christophe Verbist, Michele Giugliano

**Affiliations:** ^1^Molecular, Cellular, and Network Excitability Laboratory, Department of Biomedical Sciences, Faculty of Pharmaceutical, Biomedical and Veterinary Sciences, Institute Born-Bunge, Universiteit Antwerpen, Wilrijk, Belgium; ^2^Neuroscience Area, Scuola Internazionale Superiore di Studi Avanzati (SISSA), Trieste, Italy

**Keywords:** noise, spike-triggered average, interneuron, layer 1 cortex, dynamical transfer function

## Abstract

The cortical layer 1 (L1) contains a population of GABAergic interneurons, considered a key component of information integration, processing, and relaying in neocortical networks. In fact, L1 interneurons combine top–down information with feed-forward sensory inputs in layer 2/3 and 5 pyramidal cells (PCs), while filtering their incoming signals. Despite the importance of L1 for network emerging phenomena, little is known on the dynamics of the spike initiation and the encoding properties of its neurons. Using acute brain tissue slices from the rat neocortex, combined with the analysis of an existing database of model neurons, we investigated the dynamical transfer properties of these cells by sampling an entire population of known “electrical classes” and comparing experiments and model predictions. We found the bandwidth of spike initiation to be significantly narrower than in L2/3 and 5 PCs, with values below 100 cycle/s, but without significant heterogeneity in the cell response properties across distinct electrical types. The upper limit of the neuronal bandwidth was significantly correlated to the mean firing rate, as anticipated from theoretical studies but not reported for PCs. At high spectral frequencies, the magnitude of the neuronal response attenuated as a power-law, with an exponent significantly smaller than what was reported for pyramidal neurons and reminiscent of the dynamics of a “leaky” integrate-and-fire model of spike initiation. Finally, most of our *in vitro* results matched quantitatively the numerical simulations of the models as a further contribution to independently validate the models against novel experimental data.

## Introduction

Layer 1 (L1) is the most superficial neocortical layer and holds a key role in the hierarchy of information processing within neocortical networks. It contains a resident population of interneurons, which are solely GABAergic in the mature neocortex (Hestrin and Armstrong, 1996; Gentet, 2012). They receive afferents from a variety of brain areas, including primary and higher-order thalamic relays, cortico-cortical projections, as well as neuromodulatory afferents from subcortical structures. Thanks to this convergence, it was suggested that L1 interneurons might integrate top–down information with feedforward sensory inputs, filter out the noise in the incoming signals, and convert them into local inhibition (Larkum, 2013; Schuman et al., 2019). From L1, information is then transferred to pyramidal cells (PCs) of layer 2/3 (L2/3) and of layer 5 (L5) *via* two distinct microcircuits, which can either promote or inhibit the generation of dendritic spikes (Jiang et al., 2013). Hence, L1 interneurons may play a pivotal role in modulating, in a state-dependent manner, the coincidence detection mechanism that ensures the amplification and the further processing of attentional signals by PCs (Larkum and Zhu, 2002; Zhu and Zhu, 2004; D’Souza and Burkhalter, 2017).

However, as opposed to L2/3 and L5 PCs, the excitable properties of interneurons have not yet been examined systematically in terms of dynamical firing regimes (but see Linaro et al., 2019; Merino et al., 2019). Within neocortical networks of L2/3 and L5, we already know that information encoding and transfer feature wide-bandwidth dynamics. These performances are ensured by the rapid-onset dynamics of action potentials (APs), which allow neuronal populations to collectively phase-lock their instantaneous firing rate to the fast-varying Fourier components of the input signals (200–1,000 cycle/s; Goriounova et al., 2018; Linaro et al., 2018). In analogy to electrical filters, the upper limit to such broad neuronal bandwidth is referred to as *cut off frequency* (Brunel et al., 2001; Fourcaud-Trocmé et al., 2003), which has been experimentally measured in L2/3 and L5 pyramidal neurons upon identification of the *dynamical transfer function* of those cells (Kondgen et al., 2008; Boucsein et al., 2009; Ilin et al., 2013; Goriounova et al., 2018; Linaro et al., 2018).

For L1, we know that several subpopulations of L1 interneurons can be distinguished on the basis of their firing in response to constant amplitude currents (Muralidhar et al., 2013), displaying quite heterogeneous electrical phenotypes. Nonetheless, it is still not clear how these different electrical signatures contribute to the distinct properties in the network dynamics of information processing within L1.

In this work, by means of whole-cell patch-clamp recordings in rodent acute brain tissue slices, we examined L1 interneurons *in vitro* and identified their electrical phenotype as well as their dynamical transfer function. We quantified how L1 cells’ firing output is influenced by a temporal modulation of their input, namely, described in the Fourier domain, the cells’ filter properties of incoming input signals. Allowing a comparison with previous studies in principal cells, we specifically adopted a simple and established experimental protocol (Higgs and Spain, 2009; Ilin et al., 2013). This is equivalent (Tchumatchenko and Wolf, 2011) to our previous probing strategy of the dynamical excitable properties of cortical neurons (Kondgen et al., 2008; Linaro et al., 2018). Importantly, we showed earlier that cut off frequency and bandwidth are features that are independent on the parameters of the injected currents and the firing regimes (Linaro et al., 2018).

Given the impact that L1 interneurons have on the output of PCs, characterizing their dynamical response properties is highly relevant and timely to clarify how information integration, processing, and transfer take place to select behaviorally relevant signals. Finally, a set of previously released multicompartmental mathematical models of L1 interneurons (Markram et al., 2015) was studied under the same stimulation protocols *in vitro*, aiming at further validating them and at supporting the interpretation of the experimental data.

## Materials and Methods

### Brain Tissue Slice Preparation

Experiments were performed as described previously (Arsiero et al., 2007; Kondgen et al., 2008) and in accordance with international and institutional guidelines on animal welfare. All procedures were approved by the Ethical Committee of the University of Antwerp (permission no. 2011_87) and licensed by the Belgian Animal, Plant, and Food Directorate-General of the Federal Department of Public Health, Safety of the Food Chain, and the Environment (license no. LA1100469).

Fourteen- to twenty-one days old Wistar rats of either sex were anesthetized using isoflurane and decapitated. The brains were rapidly extracted and immersed in bubbled ice-cold artificial cerebrospinal fluid (ACSF) containing (in mM) 125 NaCl, 25 NaHCO_3_, 2.5 KCl, 1.25 NaH_2_PO_4_, 2 CaCl_2_, 1 MgCl_2_, and 25 glucose saturated with 95% O_2_ and 5% CO_2_, with pH of 7.3 and osmolarity of ~315 mOsm. Then, 300-μm-thick parasagittal slices were cut from the primary somatosensory cortex using a vibratome (VT1000 S, Leica Microsystems GmbH, Germany) and incubated in ACSF at 36°C for 30 min.

After recovery, the slices were stored in ACSF at room temperature in a holding chamber until the recordings were started. Once placed in the recording chamber, constituting the stage of an upright microscope, the L1 cells were visualized with infrared differential interference contrast microscopy under ×40 magnification. All experiments were performed in submerged conditions at a temperature of 32°C under continuous perfusion with oxygenated ACSF.

### Electrophysiology

Layer 1 cells were selected on the basis of their distance from the pia mater and from the border with L2/3, which was identified as an increase in the density of cell somata, located approximately 100 μm away from the pia mater. Within this region, the whole-cell patch-clamp configuration was established from the cell soma and the neuronal response properties were probed in the current-clamp mode. Filamented borosilicate glass pipettes were prepared using a micropipette horizontal puller (P-97, Sutter Instruments, Novato, CA, USA) and had a resistance of 4–7 MΩ when filled with an intracellular solution containing (in mM) 115 K-gluconate, 20 KCl, 10 4-(2-hydroxyethyl)-1-piperazineethanesulfonic acid, 4 adenosine triphosphate-Mg, 0.3 Na_2_-guanosine triphosphate, and 10 Na_2_-phosphocreatine, with the pH adjusted to 7.3 with KOH and osmolarity of ~290 mOsm. Recordings and intracellular current stimulation were performed using an Axon Multiclamp 700B Amplifier (Molecular Devices, San Jose,CA, USA) controlled by a personal computer running a real-time Linux operating system (Linaro et al., 2014). For more information on how to install our real-time software, see Linaro et al. (2015). The recorded voltage waveforms were sampled at a frequency of 30 kHz and digitized at 16 bit. In order to compensate for the glass pipette electrical resistance and capacitance, a digital non-parametric model was repeatedly identified throughout the recording sessions by a computer-aided technique, known as *active electrode compensation* (Brette et al., 2008). This allowed us to digitally separate the electrode and the membrane contributions to the recorded traces, requiring neither the bridge balance nor the capacitance neutralization circuits of the amplifier. The adoption of such a technique became a routine procedure in our laboratory for both conventional and real-time experiments (Couto et al., 2015; Linaro et al., 2018, 2019). However, while for the dynamic clamp online accurate “active” electrode compensation is necessary to avoid recording instabilities (Brette et al., 2008; Linaro et al., 2019), for the current-clamp—in the context of the present work—it is not strictly required. In fact, on one hand, we focused here on probing the dynamical transfer function of neurons in the current-clamp mode by spike-triggered averaging (see below) so that accurate *active electrode compensation* was not imperative. Indeed despite using a single electrode for both stimulation and recordings, only the times of AP occurrence must be detected for further analysis (see below). Such detection occurs, by definition, with a very high signal-to-noise ratio and it usually does not represent a problem, even with imperfect electrode compensation. On the other hand, estimating the features of AP waveforms definitely benefits of a more accurate (non-parametric) compensation procedure than the (parametric) one allowed by the electronic amplifier controls. Finally, postponing the compensation of all acquired traces to an offline automated procedure ultimately offered us an efficient management of time during each experiment, while only requiring to periodically run a “calibration” protocol (Linaro et al., 2015).

### Electrical Phenotype Identification

The recorded voltage traces were processed and analyzed offline in MATLAB (The MathWorks, Natick, MA, USA). Data from *N* = 65 L1 interneurons were included in this study, which were selected on the basis of a healthy cell resting membrane potential (≤65 mV) and AP peak amplitude (>50 mV). These criteria were considered to be indicative of a good patch stability and proper electrical access to the cell. The membrane input resistance, capacitance, and time constant were estimated by standard procedures (Kondgen et al., 2008). Briefly, hyperpolarizing current steps of decreasing amplitudes [i.e., (−200; 0) *pA* lasting 1 s each] were repeatedly applied and the voltage response was recorded. The membrane input resistance was then identified as the slope of the best-fit straight line to the steady-state data points in the voltage vs. current plane. The membrane time constant was instead extracted as the slowest time constant of the best-fit bi-exponential function, describing the recovery of the membrane potential from 10–ms -long hyperpolarizing pulses of amplitude −150 pA. The cell capacitance was finally estimated as the ratio between the time constant and the input resistance of the membrane.

**Figure 1:**
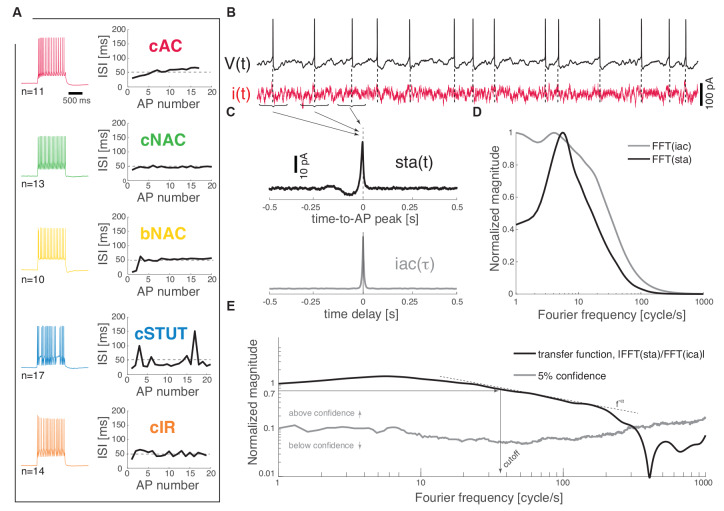
Dynamical transfer function identification in L1 cortical interneurons. Distinct subtypes of L1 interneurons can be distinguished **(A)** from their electrical response pattern by the sequence of interspike intervals in a 20 spike/s train. These are continuous accommodating, c. non-accommodating, bursting non-accommodating, c. stuttering, and c. irregular. Regardless of their identity, the neurons were stimulated by a fluctuating current *i*(*t*; **B**) while recording their voltage response *V*(*t*). The spike-triggered average [i.e., *sta*(*t*)] of the stimulus and its autocorrelation function [i.e., *iac*(*τ*)] were computed **(C)** and fast Fourier-transformed **(D)** to estimate the neuron dynamical transfer function **(E)**. As in electronic filters, the magnitude of this function expresses the intensity of the output firing rate of the cell across temporal modulations or Fourier components of an input signal, thus revealing the bandwidth of spike initiation dynamics. The cut off was characterized—above significance (see “Materials and Methods” section)—as the frequency corresponding to a 70% decrease of the response magnitude of the value taken at 1 cycle/s. The high-frequency profile was finally described as a *f*^−α^ power-law.

Each recorded neuron was classified in one of the five identified subtypes on the basis of their response to depolarizing current pulses (Muralidhar et al., 2013). Briefly, a *frequency*–*current* curve was first computed upon injecting current steps of increasing depolarizing amplitudes [i.e., in the range (0; 300) pA, lasting 1 s]. The voltage responses containing a train of APs, corresponding to a mean firing rate of 20 spike/s, were compared to each other as the sequence of successive inter-spike intervals (ISIs) was plotted (Figure 1A). Sorting each cell into one of the five classes (i.e., cAC, continuous accommodating; cNAC, continuous non-accommodating; bNAC, bursting non-accommodating; cSTUT, continuous stuttering; and cIR, continuous irregular firing) was performed manually, following closely (Muralidhar et al., 2013) and according to the following criteria: cAC, if the slope of the best-fit straight line over the ISI sequence was larger than 1 ms; cNAC, if the best-fit line was mostly horizontal (i.e., slope smaller than 1 ms); bNAC, if the initial 1 to 2 ISIs were shorter than 20 ms and followed by a train of APs showing no accommodation; cSTUT, if at least one ISI was equal or larger than 100 ms; and cIR, if the ISI sequence was irregular, with individual values shorter than 100 ms. The above criteria led to classifying 11 cells as cAC, 13 as cNAC, 10 as bNAC, 17 as cSTUT, and 14 as cIR (Figure 1A).

### Spike-Triggered Average and Dynamical Transfer Function

Wide-band input current waveforms *i*(*t*) were injected into the cell soma in order to probe their first-order dynamical transfer properties, following closely (Ilin et al., 2013; see also Higgs and Spain, 2009). Under these conditions, neuronal firing is irregular and subthreshold membrane potential fluctuations resemble the activity recorded *in vivo* (Destexhe et al., 2003). The injected current was defined as the sum of a DC and of a fluctuating component (Figure 1B):

(1) $$i(t)=i_{0}+\sigma \eta (t)$$

with *η*(*t*) being an independent realization of an Ornstein–Uhlenbeck stochastic process (Cox and Miller, 1965) with zero mean, unitary variance, and correlation time *τ* = 5 ms. *η*(*t*) was generated offline, iterating an algebraic expression (Gillespie, 1996).

By such a definition, σ represents the standard deviation of the noisy fluctuation in *i*(*t*), while *i*_0_ is its expected value. In each experiment, σ was adjusted to obtain membrane potential fluctuations with 4 mV standard deviation and ≈15 mV peak-to-peak changes. The value of *i*_0_ was instead chosen to maintain the mean firing rate of the neurons in the range of 3–6 spike/s. Each stimulation trial lasted 60 s and was preceded by brief current steps, monitoring over time the stability of the recording, i.e., in terms of resting potential, input resistance, and mean firing rate as in Kondgen et al. (2008). The stimulation was repeated several times, with distinct realizations of *η*(*t*), until at least 3,500 APs in total were collected, while allowing sufficient inter-stimulus recovery intervals of up to 60–100 s, depending on the cell. This resulted in 18–20 repetitions, corresponding to approximately 40–45 min of recording in total.

We recorded the train of APs fired by the cell in response to the injected stimulus *i*(*t*), evaluating offline the spike-triggered average *sta*(*t*) of such a stimulus waveform. This analysis allows an estimate of the dynamical transfer function (Kondgen et al., 2008) in biological neurons as well as model neurons and was performed following closely Ilin et al. (2013). Briefly, *sta*(*t*) was evaluated as the ensemble average of the data points of *i*(*t*) that shortly preceded and followed the peak of each AP fired, i.e., over the times *t*_1_, *t*_2_, *t*_3_, …, *t*_N_ of AP occurrences (Figures 1B,C):

(2) $$sta(t)=<\sum_{k=1}^{N}i(t_{k}-t)>t\in [-T;T]$$

where *T* = 500 ms is the chosen time interval preceding and following each AP. This expression can be equivalently rewritten as an ensemble average of the convolution between *i*(*t*) and a train of Dirac’s delta functions *s*(*t*); (i.e., one for each AP):

(3) $$sta(t)=<\int_{-\infty }^{+\infty }s(\tau )i(\tau -t)d\tau >s(t)=\sum_{k=1}^{N}\delta (t-t_{k})$$

Invoking linearity and swapping integral and average operators, we may derive another expression (Dayan and Abbott, 2005) linking *sta*(*t*) to the instantaneous firing rate *r*(*t*) associated to the AP train *s*(*t*):

(4) $$sta(t)=\int_{-\infty }^{+\infty }r(\tau )<i(\tau -t)>d\tau$$

In the Fourier domain, this convolution integral simplifies the product of the firing rate transform *R*(*f*) and the average (complex conjugate) of the input transform I*(*f*) :

(5) STA(f) = R(f)< I*(f) >

Finally, as *R*(*f*) is also the product of the first-order dynamical transfer function *H*(*f*) (Marmarelis and Naka, 1972) times the average input < *I* (*f*) > (Brunel et al., 2001),

(6) R(f) = H(f)< I(f) >

the transfer function *H*(*f*) (Figure 1E) can be computed as the ratio between the *sta*(*t*) (fast) Fourier transform and the power spectral density of *i*(*t*) (Figures 1C,D):

(7) H(f) = STA(f)/<I(f)I*(f) > 

where the power spectral density of *i*(*t*) is the (fast) Fourier transform of its autocorrelation function *iac*(τ) = < *i*(*t*)*i*(*t*−τ)>.

The profile of the transfer function was considered above the 5% confidence threshold (Figure 1E) generated by a bootstrap method on surrogate data (Press, 2007). Briefly, these were obtained upon generating 500 times a random shuffling of the original interspike intervals and repeating each time the *sta*(*t*) analysis in the Fourier domain. The confidence threshold was then computed at each frequency as the sum of the mean (surrogate) transfer function (i.e., over the 500 surrogate trials) and its (surrogate) standard deviation.

The *cut off* frequency was then defined as the frequency at which the magnitude of the transfer function ||*H*(*f*)|| (above the confidence threshold) decreases down to 70% of the value it takes at 1 Hz (Figure 1E).

For very large Fourier frequencies *f*, the magnitude of the transfer functions decayed as a negative power-law, i.e., *f*^−α^ (Fourcaud-Trocmé et al., 2003; Kondgen et al., 2008; Linaro et al., 2018). In order to best describe the input–output transformation of the neurons in this high spectral domain, the part of the transfer curves going from the cut off frequency down to 20% of the cut off value was fitted by a power-law *y* = *bx*^−α^ where α describes the slope of the decay in log–log coordinates.

### Rapidness of the Action Potential at Its Onset

The average waveform of the AP was examined for each cell by averaging the APs fired during the steady-state response regime of the recorded voltage responses. The threshold for AP initiation (in mV) was conventionally calculated as the potential where the change in voltage over time is 20 mV/ms (Naundorf et al., 2006). When plotted in the plane *dV*_m_/*dt* vs. *V*_m_, each AP described a closed trajectory. The AP speed at onset (expressed in ms^−1^) was then measured in this plane as the slope of the tangent line to the AP trajectory at the voltage coordinate corresponding to the AP threshold.

The *dynamic IV curve* method was also employed to quantify the AP waveforms, relating the upstroke phase of an AP to the best-fit equation of a non-linear (i.e., exponential) relationship between *dV*_m_/*dt* and *V*_m_ (Brette et al., 2008; Badel et al., 2008a,b). From the resulting fit, the spike–slope factor *Δ*_T_ was extracted to further quantify the rapidness of the AP.

### Computer Simulations

The simulation of 69 distinct L1 interneuron models was performed in NEURON (Hines and Carnevale, 2001; Carnevale and Hines, 2006) using the publicly available Blue Brain Project (BBP) database (Markram et al., 2015). Each model was originally built from experimental data collected from L1 neurons classified into the same electrical response phenotypes employed in this work (see also Ascoli et al., 2008).

**Figure 2:**
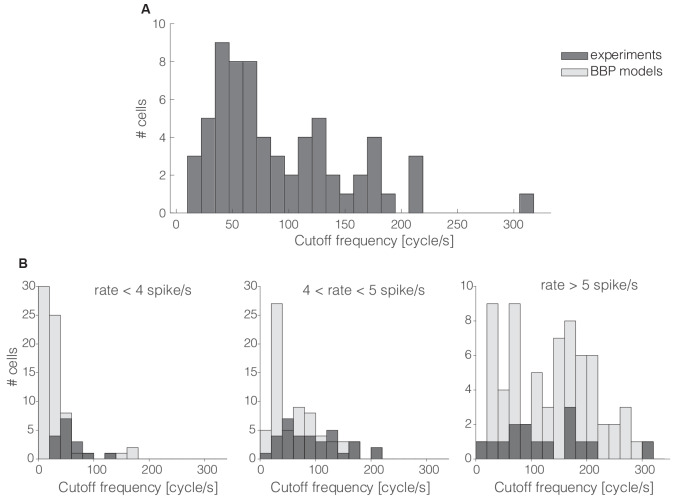
The cells’ cutt off frequency distribution. More than 60% of the 65 interneurons recorded in this work had their cut off frequencies markedly below 100 cycle/s **(A)**. The same trend was quantitatively confirmed *in silico* by repeating the same protocol as that of Figures 1B–E in 69 L1 neuron models **(B)** as released by the Blue Brain Project (BBP). Numerical simulations and experimental data were compared over three distinct ranges of experimental firing rates, where models were set to fire at precisely 3, 5, and 7 spike/s (**B**; from left to right). The histogram overlay displays the rather good model predictions of the horizontal span of cut off frequencies over the three distinct firing rates.

As close as possible, we mimicked *in silico* the very same stimulation protocols and analysis employed *in vitro*. For the spike-triggered average estimate, we chose the parameters of the injected current *i*(*t*) by means of an iterative procedure based on the bisection method (Press, 2007). Given the increased reproducible character of simulated neuronal responses compared to experiments, by selecting *i*_0_ we could set the firing rate of the models with higher precision. We then chose three regimes (3, 5, and 7 spikes/s) to cover the entire range obtained in our experiments (3–6 spike/s) with increased confidence. We repeated 60 s-long stimulations *in silico* until a minimum of 5,000 APs were collected, and we followed closely the analysis methods described in the previous sections (Figure 2).

### Statistical Analysis

**Figure 3:**
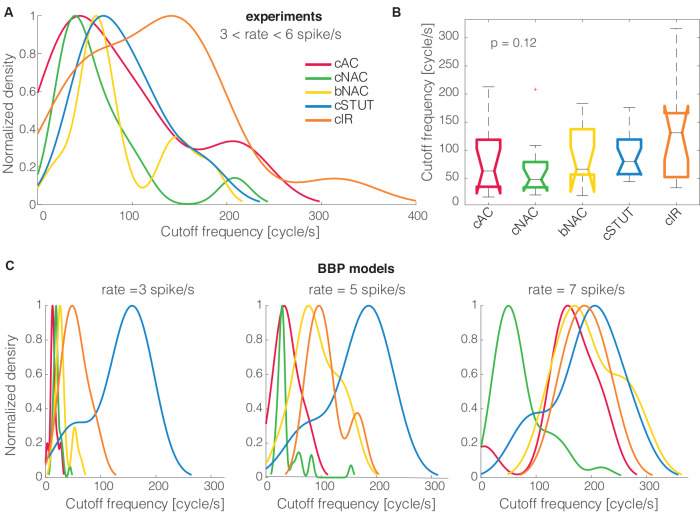
The cells’ electrical phenotype and cut off frequency distributions. When the five distinct electrical classes of L1 interneurons were compared to each other, their (smoothed) probability density distributions of the cut off frequencies, normalized by their peak values, showed no major differences **(A)**. Quantitatively, the Kruskal–Wallis statistical test failed to reject, at 1% significance level, the null hypothesis that the cut off frequencies come from the same distribution **(B)**. A similar trend was largely reproduced *in silico* for the BBP models **(C)**.

All numerical data are presented as mean ± standard deviation. A statistical analysis of all correlations between parameters was performed using the Pearson correlation test (Press, 2007), thus reporting the values of the correlation coefficient *ρ* and its *p*-value. The one-way analysis of variance by Kruskal–Wallis was employed [i.e., the MATLAB kruskalwallis() command] to reject the hypothesis, at 1% significance, that the observables extracted from distinct electrical phenotypes originate from the same probability density distribution. Finally, qualitative comparison between the distribution densities of cut off frequencies, among different electrical phenotypes (Figure 3), were performed, normalizing the peak amplitudes of smoothed histograms by the kernel smoothing method [i.e., the MATLAB histfit() command].

## Results

We describe the firing response properties of L1 cortical interneurons based on a set of whole-cell patch-clamp recordings in *N* = 65 cells from slices of the rat somatosensory cortex. We studied systematically both passive and active membrane properties, revealing that cells had an input resistance of 184.40 ± 51.15 MΩ a membrane capacitance of 201.51 ± 62.95 nF, and a membrane time constant of 35.77 ± 10.23 ms (see “Materials and Methods” section). When active response properties were studied, we identified distinct electrical phenotypes and sorted the cells into five separate classes (Muralidhar et al., 2013). Such a classification was based on the analysis of the time course of the ISIs sequence, during ≈ 20 spike/s trains of APs in response to a current step lasting 1 s (Figure 1A).

### Encoding Properties of L1 Interneurons

We studied the encoding properties of L1 interneurons by measuring their *dynamical transfer function* in the Fourier domain (Figures 1B–E). Following closely Ilin et al. (2013), we injected randomly fluctuating current stimuli into the soma of the cells, mimicking the irregular and intense synaptic activity present *in vivo* in an intact cortex. A DC offset was also superimposed to the injected current, with its amplitude adapted so that the output mean firing rate was in the range 3–6 spike/s. Under these conditions (see “Materials and Methods” section), cells fired irregularly (Figure 1B) with a coefficient of variation of 0.53 ± 0.09 for their distribution of ISIs. This stimulation protocol enabled us to measure the spike-triggered average waveform of the injected current *sta*(*t*) and compare it to its autocorrelation function *iac*(*t*) in the Fourier domain (Figures 1C,D). In fact, the ratio of the two quantities in the transformed domain immediately leads to an estimate of the dynamical transfer function (Figure 1E; see “Materials and Methods” section) arising from the cell’s spike initiation mechanisms.

The magnitude and the phase of the transfer function, especially for large spectral frequencies, allow one to predict the collective dynamics of a neuronal population in response to rapid external inputs (Fourcaud-Trocmé et al., 2003; Kondgen et al., 2008) as well as to interpret oscillatory regimes (Wang, 2010). Here, we quantified the bandwidth of the neuronal transfer function, expressing the numerical value of the conventional high-frequency *cut off* limit (see “Materials and Methods” section). The distribution of the *cut off* frequencies demonstrates that, in general, L1 interneurons can encode fast-varying input signals up to 200 cycles/s (Figure 2A). However, differently from L2/3 and L5 PCs (Testa-Silva et al., 2014; Linaro et al., 2018), the majority (i.e., ≈65%) of L1 neurons unexpectedly display a *cut off* below 100 cycles/s.

These observations were confirmed *in silico* by applying the experimental protocol of Figures 1B–E on a large public database of 69 detailed multicompartmental models of rat cortical L1 interneurons (see “Materials and Methods” section). At a reference mean firing rate of 5 spikes/s, around 78% of the models display a *cut off* frequency below 100 cycles/s. Figure 2B compares experimental and simulated data when the computer models were set to fire on average at (from left to right) 3, 5, and 7 spikes/s. Under these conditions, the coefficients of variation of their respective ISI distributions were 0.74 ± 0.12, 0.65 ± 0.12, and 0.58 ± 0.12, respectively, for 3, 5, and 7 spikes/s mean firing rates. Real cells were thus found to be in good agreement with the range of *cut off* frequencies displayed by the models when their firing rate was sorted in close intervals.

### Electrical Classes and Encoding Properties

When the cells’ electrical classes were explicitly taken into consideration, we found no preference in the *cut off* frequency distributions (Figures 3A,B). Indeed the probability distribution densities for each group of continuous accommodating (cAC), c. non-accommodating (cNAC), bursting non-accommodating (bNAC), c. stuttering (cSTUT), and c. irregular (cIR) showed a substantial overlap (Figure 3A) and no significant differences were found (Figure 3B).

The same analysis was repeated *in silico*, where the diversity of electrical (as well as morphological) phenotype is made explicit *a priori* by a distinct set of electrotonic and excitable properties (Markram et al., 2015). The model’s electrical identity played a role in shaping, to some extent, the probability distribution densities of the cSTUT and the cNAC classes in particular.

### Dependency of *Cut Off* on the Mean Firing Rate

**Figure 4:**
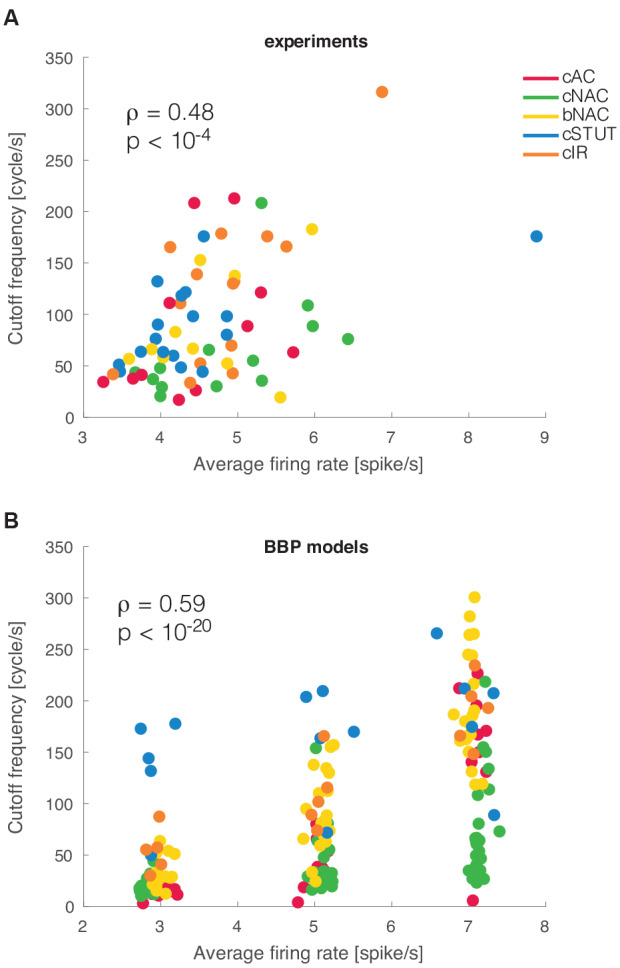
Mean firing rate modulation of the cut off frequencies. As suggested by earlier theoretical studies, a significant correlation was observed between the mean firing rate of a cell and the cut off frequency of its dynamical transfer function **(A)**. The very same result was reproduced *in silico* for the BBP models **(B)**.

Previous theoretical investigations on the dynamical properties of spike initiation reported that, in integrate-and-fire neuron models, the *cut off* frequency is sensitive to the mean firing rate. Thus, the higher the rate, the wider the bandwidth (Brunel et al., 2001; Fourcaud-Trocmé et al., 2003). This was examined experimentally in L5 PCs but failed to be confirmed as the mean firing rate altered the dynamical transfer function at low, not high, spectral frequencies (Linaro et al., 2018). Despite the small range of our experimental firing rates, we asked whether L1 interneurons behave differently than PCs. We thus studied the correlation between *cut off* frequency and the mean firing rate and found it to be very significantly correlated (*ρ* = 0.48 and *p* < 0.001), as also exemplified in Figure 4A. This implies that the boundary in the Fourier domain, where fast input signal components get filtered out, increases considerably even with a moderate increase in the neuronal firing rate.

When the analysis was repeated *in silico*, a similar phenomenon was replicated for the entire population of multicompartmental neuron models. There we found a similar correlation with a much stronger significance (*ρ* = 0.58 and *p* < 10^−20^; Figure 4B). This effect is very apparent in the simulated experiment reported in Figure 4B as most electrical types progressively shift their *cut off* frequency upwards for increasing firing rates. Such a susceptibility was further quantified by linear fitting the simulated data points at different firing rates for each firing type. The slope of this fit was 34.57 cycles/spike for cAC, 11.96 for cNAC, 41.16 for bNAC, 11.43 for cSTUT, and 32.78 for cIR. This indicates that cSTUT and cNAC models were the least sensitive to their firing rate, while cAC, bNAC, and cIR were the most sensitive.

### Action Potential Rapidity at Onset

The broad bandwidth of the AP initation dynamics has been related, in both theoretical (Fourcaud-Trocmé et al., 2003) and experimental works (Testa-Silva et al., 2014; Linaro et al., 2018), to the rapidity of APs at their onset. Thus, neurons with fast AP onset dynamic keep track of the most rapid spectral components in the input signals, as demonstrated for L2/3 and L5 PCs (see also Goriounova et al., 2018). However, when we examined the correlation between AP rapidity and *cut off* frequency in L1 inteneurons, we failed to confirm the previous reports. In fact, correlations were not significant (*ρ* = 0.13 and *p* = 0.32).

As we repeated the analysis *in silico* for all L1 model neurons available, we also observed a lack of significant correlations as in the experiments (*ρ* = 0.15 and *p* = 0.22, *ρ* = 0.08 and *p* = 0.32, and *ρ* = 0.16 and *p* = 0.20 at firing rates of 3, 5, and 7 spikes/s, respectively). Moreover, no clear separation of AP rapidity at onset was found across the electrical classes of the model cells. Even when the impact of some morphological features was considered (as in Eyal et al., 2014; Goriounova et al., 2018), we found no significant correlations between the AP onset rapidity and the total dendritic length of the model cells over a broad range of total dendritic lengths 500–5,400 μm (*ρ* = 0.11 and *p* = 0.38, *ρ* = 0.10 and *p* = 0.40, and *ρ* = 0.11 and *p* = 0.35 at firing rates of 3, 5, and 7 spikes/s, respectively). However, unexpectedly, we found slightly significantly negative correlations between the total dendritic length and the *cut off* frequency (*ρ* = −0.3 and *p* = 0.011, *ρ* = −0.31 and *p* = 0.011, and *ρ* = 0.10 at a firing rate of 3, 5, and 7 spikes/s, respectively).

As the last result was neither anticipated in simulation studies (Eyal et al., 2014) nor matched with others experimental reports, we further characterized *in vitro* and *in silico* the AP initiation employing the *dynamic IV curve* (Badel et al., 2008a,b). This allowed us to extract an additional quantitative parameter for AP initiation, known as the slope factor *Δ*_T_ (see “Materials and Methods” section). Across all our experiments, *Δ*_T_ took values smaller than 2.5 mV (1.51 ± 0.73 mV), a range that was confirmed and replicated *in silico*, consistently with the larger spike *sharpness* of interneurons compared to PCs (Badel et al., 2008a).

While *Δ*_T_ and the AP onset rapidity showed correlations *in silico* (*ρ* = 0.26 and *p* = 0.03 at 5 spike/s), their values *in vitro* had no significant correlation (*ρ* = −0.11 and *p* = 0.37). Importantly, as the values of *Δ*_T_ and the *cut off* frequency were compared across cells, a significant correlation was finally observed in our experimental data (*ρ* = −0.31 and *p* = 0.012), but not *in silico* (*ρ* = −0.06 and *p* = 0.06 at 5 spike/s).

### Transfer at High Spectral Frequencies

**Figure 5:**
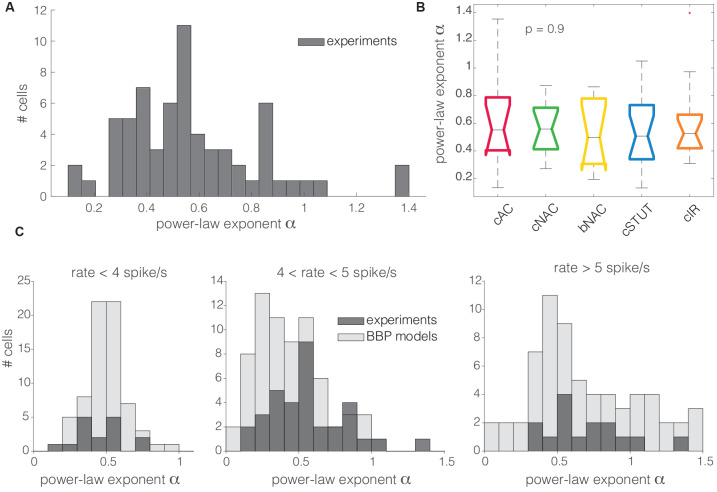
The distribution of the power-law exponent at high spectral frequencies. Almost all the cells recorded in this work had a power-law exponent lower than 1 for high spectral frequencies **(A)**. Concerning the cell subtypes, the same as for Figure 3B, the Kruskal–Wallis statistical test failed to reject at, 1% significance level, the null hypothesis that the cut off frequencies come from the same distribution **(B)**. The same trend was quantitatively confirmed *in silico* for the BBP models **(C)**. Numerical simulations and experimental data were compared over three distinct ranges of experimental firing rates, where the models were set to fire at precisely 3, 5, and 7 spike/s (**C**; from left to right). The histogram overlay displays the model predictions of the horizontal span of the cut off frequencies over the three distinct firing rates.

For high spectral frequencies *f* (i.e., above the *cut off*), the dynamical transfer function is known to decay as *f*^−α^ (Kondgen et al., 2008), where the numerical value of the exponent has been linked to the precise dynamics of AP initiation at threshold (Fourcaud-Trocmé et al., 2003). In comparison with standard simplified models of excitability, such as the integrate-and-fire units (Tuckwell, 1988), previous results in pyramidal neurons [i.e., *α* ∈ (1;1.5)] consistently pointed towards an *exponential* or *polynomial* dependency of the AP initiation on the membrane potential (Kondgen et al., 2008; Linaro et al., 2018). Unexpectedly, as we analyzed the transfer properties of L1 neuron at high spectral frequencies, we found values of α in the range from 0 to 1.4, with the largest majority (i.e., 94%) smaller than 1 (0.57 ± 0.26; Figure 5A). The distribution of α across different electrical cell types displayed largely overlapping features (Figure 5A) with no significant differences, thus hinting at similar transfer behaviors.

These observations were confirmed *in silico*, where L1 interneuron models were characterized by α in the range 0.2–1.5, with the majority below 1 (0.51 ± 0.14, 0.44 ± 0.24, and 0.74 ± 0.43 at firing rates of 3, 5, and 7 spike/s, respectively), matching the values obtained in the experiments (Figure 5B).

## Discussion

In this work, we have examined the dynamical signal transfer properties of L1 cortical interneurons. These cells are likely to play a major role in cortical computation as they receive several afferents from a variety of brain regions while establishing synapses downstream in several cortical columns. Their function of gating and filtering the output spike trains of PCs in other layers appears clear, although its careful spectral characterization remained so far unexplored. When an established protocol (Figure 1) was used to quantify the encoding properties of L1 interneurons, a markedly lower bandwidth (Figure 2) and a gentler attenuation at high spectral frequencies were observed (Figure 5), compared to L2/3 and L5 PCs. Thus, while the rapidly varying frequency component of their inputs can be tracked, i.e., up to 200–300 cycle/s, the large majority of L1 interneurons possesses much lower *cut off* values, below 100 cycle/s. This might indicate that L1 outputs may be well suited to filter incoming information and relay it to PCs, especially in the center of their own bandwidth (Ilin et al., 2013; Goriounova et al., 2018; Linaro et al., 2018), particularly where the phase delay introduced by the PCs transfer function is minimal (Linaro et al., 2018).

The markedly lower *cut off* frequencies and smaller power-law attenuation coefficient α in L1 interneurons might therefore indicate their specialization at keeping track of slower *top*–*down* input modulations (Jiang et al., 2013; Larkum and Phillips, 2016) compared to *bottom*–*up* inputs reaching directly PCs in L2/3 and L5, while attenuating less rapidly the components at higher spectral frequencies. Despite the limited size of our data set, we successfully sampled all the known electrical subtypes of L1 cells and found no significant differences in their spectral responses (Figure 3). This finding is backed up by an extensive database of accurate multicompartmental models (Markram et al., 2015) that contains *a priori* an extensive diversity in cell response properties.

Our choice of the *in vitro* protocol and the limited cell viability during long experimental conditions did not allow us a systematic exploration of the modulatory effect of the cell’s mean firing rate on the neuronal bandwidth. Nonetheless, we could establish that a significant correlation exists between the *cut off* frequency and the cell’s firing rate (Figure 4), as anticipated by the theory (Brunel et al., 2001; Fourcaud-Trocmé et al., 2003) but not previously reported for PCs (Linaro et al., 2018). This suggests that an increase in the mean firing rate of L1 interneurons can expand their filtering capabilities, perhaps relaying to the dendritic compartment of PCs distinct spectral information during different cortical firing regimes. The simulations also suggest that some neuronal types should be more susceptible for the firing rate modulation, such as cAC, bNAC, and cIR types.

As opposed to previous reports in PCs, when we characterized the AP waveform and its relevance for the tracking of fast-input spectral components, we did not observe any correlation with the values of the *cut off* frequency, both *in vitro* and *in silico*. Therefore, we suggest that the emerging mechanisms for spike initiation of L1 interneurons might have some quantitative differences when compared to those of excitatory neurons (Ilin et al., 2013; Linaro et al., 2018). In contrast with other studies (Goriounova et al., 2018), we found a negative correlation between the total dendritic length and the cut off frequency *in silico*. This might be a consequence of the smaller dendritic length in L1 models than L5 PCs (i.e., 500–5,400 vs. 8,000 μm and up to 15,000–20,000 μm), although the values of AP onset speed were in a similar range as for L5 PCs, both in experiments and in simulations. No correlation was found between the AP onset speed and the total dendritic length in the models. However, as the AP slope factor *Δ*_T_ was measured, we found similar values as those obtained for other neocortical types. *In silico*, an (expected) negative correlation was found between *Δ*_T_ and the AP onset rapidity (Badel et al., 2008a,b), but not *in vitro*. A correlation between *Δ*_T_ and the *cut off* frequency clearly was detected *in vitro*, but not *in silico*. We speculate that such a mismatch between theory and experiments might be a consequence of the reduced repertoire of active membrane mechanism models (i.e., sodium and potassium currents kinetics), which are shared by all BBP cortical model neurons (Markram et al., 2015). While using the same biophysical models for describing all cell types is a convenient strategy for constraining automated parameters (Druckmann et al., 2007), we wonder whether L1 interneurons might be even better described with “custom” kinetic parameters for sodium and potassium currents. In addition, we think that the excitability of L1 interneurons has been investigated partially and in less detail when compared to L5 PCs. The latter have been widely studied as a “reference” cortical neuron by many investigators over the last decades. Lastly, BBP L1 models have been identified automatically while extracting only a limited set of features from the experimental data (e.g., time-to-first AP, width of the AP, AP frequency, etc.) (Druckmann et al., 2008), and we wonder whether the use of additional protocols, such as the probing of the dynamical transfer function, might have increased the faithfulness of theory–experiments matching. Taken together, all these results call for further investigation of the AP initiation mechanisms in L1 interneurons, both experimentally and numerically.

Finally, as we characterized the transfer properties at high spectral frequencies, we observed a power-law decay, although with unexpectedly low absolute values of the exponent α, both *in vitro* and *in silico*. According to Fourcaud-Trocmé et al. (2003), different reduced models of excitability are associated to distinct characteristic values of α. In particular, while the exponential integrate-and-fire unit seems to be more appropriate to describe PCs, the leaky integrate-and-fire excitability (i.e., *α* = 0.5) seems closer to explain the data, both *in vitro* (i.e., *α* = 0.57 on average) and *in silico* (i.e., in the range *α* = 0.44–0.74). This suggests that L1 interneurons indeed might have distinct spike initiation dynamics when compared to PCs.

In conclusion, we believe that our results contribute with timely and relevant observations to the series of efforts, worldwide, to describe and classify the excitable properties of neocortical neurons. The spectral characterization of the bandwidth of spike initiation thus revealed to be more informative than the standard methods to quantify neuronal excitability, and the present study extends such characterization to the population of L1 interneurons.

## Data Availability Statement

Relevant datasets and analysis scripts are available at FigShare.com (https://dx.doi.org/10.6084/m9.figshare.12091047).

## Ethics Statement

The animal study was reviewed and approved by Ethical Committee of the University of Antwerpen (permission n. 2011_87) and Belgian Animal, Plant, and Food Directorate-General of the Federal Department of Public Health, Safety and Food Chain and the Environment (license n. LA1100469).

## Author Contributions

MG conceived and designed the research. SB performed the experiments. CV performed the simulations. SB and CV analyzed the data. SB, CV, and MG wrote the article.

## Conflict of Interest

The authors declare that the research was conducted in the absence of any commercial or financial relationships that could be construed as a potential conflict of interest.
